# *PTEN* Redundancy: Overexpressing *lpten*, a Homolog of *Dictyostelium discoideum ptenA*, the Ortholog of Human *PTEN*, Rescues All Behavioral Defects of the Mutant *ptenA^−^*

**DOI:** 10.1371/journal.pone.0108495

**Published:** 2014-09-23

**Authors:** Daniel F. Lusche, Deborah Wessels, Nicole A. Richardson, Kanoe B. Russell, Brett M. Hanson, Benjamin A. Soll, Benjamin H. Lin, David R. Soll

**Affiliations:** Monoclonal Antibody Research Institute and Developmental Studies Hybridoma Bank, Department of Biology, The University of Iowa, Iowa City, Iowa, United States of America; Aristotle University of Thessaloniki, Greece

## Abstract

Mutations in the tumor suppressor gene *PTEN* are associated with a significant proportion of human cancers. Because the human genome also contains several homologs of *PTEN*, we considered the hypothesis that if a homolog, functionally redundant with PTEN, can be overexpressed, it may rescue the defects of a *PTEN* mutant. We have performed an initial test of this hypothesis in the model system *Dictyostelium discoideum*, which contains an ortholog of human *PTEN*, *ptenA*. Deletion of *ptenA* results in defects in motility, chemotaxis, aggregation and multicellular morphogenesis. *D. discoideum* also contains *lpten*, a newly discovered homolog of *ptenA.* Overexpressing *lpten* completely rescues all developmental and behavioral defects of the *D. discoideum* mutant *ptenA^−^*. This hypothesis must now be tested in human cells.

## Introduction

*PTEN* is one of the most commonly mutated tumor suppressor genes in the progression of human cancers [1]–[3]. It has been demonstrated to play a prominent role in several cellular behaviors, including basic cell motility, chemotaxis and invasion [4]–[10]. PTEN functions as a phosphatase that regulates the signal transduction molecule phosphatidylinositol-3, 4, 5-triphosphate (PIP_3_) [11]. There are three homologs of the *PTEN* gene in the human genome [12]–[16]. In addition, Poliseno et al. (2010) [17], [18] found that a *PTEN* pseudogene, *PTENP1*, regulates the level of PTEN protein and acts as a growth suppressor. The presence of *PTEN* homologs in the human genome, therefore, raises the possibility that one of them may be able to substitute functionally for a mutated *PTEN* under inducing conditions, thus suppressing tumorigenesis, a possibility heretofore not tested.

The amoeba *Dictyostelium discoideum,* an exceptional model for studying the regulation of human cell motility and chemotaxis [19]–[27], contains the gene *ptenA*, an ortholog of the human *PTEN* gene. Deletion of *ptenA* in *D.discoideum* causes major defects in lateral pseudopod suppression, motility, chemotaxis and natural aggregation [28]–[34]. As is the case for human PTEN, PtenA in *D.discoideum* dephosphorylates phospahtidylinositol (3,4,5)-trisphosphate (PIP_3_) to form phophatidylinositol (4,5)-bisphosphate (PIP_2_) [35], [36] and mediates PIP_3_ oscillations [37]–[41], which correlate with actin polymerization and pseudopod extension [30], [39]–[43]. PtenA was originally thought to be the sole phosphatase for the dephosphorylation of PIP_3_ to PIP_2_ in *D. discoideum*. However, after global stimulation of *ptenA^−^* cells with the chemoattractant cAMP, the concentration of PIP_3_ increases, but then declines [36], indicating that PIP_3_ is degraded to PIP_2_ in the absence of PtenA, presumably by another phosphatase. Moreover, Hoeller and Kay [32] demonstrated that when suspensions of *ptenA^−^* cells were pulsed with cAMP to induce chemotactic responsiveness, they were able to undergo efficient chemotaxis. However, unlike earlier studies in which the concentration of the cAMP gradient, generated *in vitro* was in the range of that estimated for the gradient in the front of a natural cAMP wave [44], Hoeller and Kay [32] employed a cAMP gradient generated in a concentration range 10 times higher than that employed in the prior studies of *ptenA^−^* chemotaxis [29], [30] and, therefore, 10 times higher than that estimated for the natural cAMP wave that induces chemotaxis in natural populations [44]. The studies of PIP_3_ degradation in *ptenA^−^* cells after global cAMP stimulation [36] and chemotaxis of *ptenA^−^* cells in high cAMP concentration gradients [32], suggested to us that there might be an alternative PIP_3_ phosphatase that could substitute for *ptenA*.

We therefore searched the *D.discoideum* database (http://dictybase.org/) and found a second ortholog of human *PTEN* and homolog of *ptenA* [28], [29], which we named *lpten* because it contained unique LIM domains. Here we show that cells of the *lpten* deletion mutant, *lpten^−^*, exhibit defects in behavior similar to those in *ptenA^−^* cells, but the defects are far weaker. To test for redundant function, we overexpressed *lpten* in a *ptenA^−^* background. Overexpression resulted in the complete normalization of the defective behaviors of *ptenA^−^* cells. The *ptenA^−^* defects that were normalized included the following: abnormal aggregation, the absence of multicellular morphogenesis, the loss of lateral pseudopod suppression, increased turning, decreased cellular velocity, aberrant chemotaxis in a cAMP gradient generated in the standard concentration range and aberrant natural aggregation. We further show that pulsing *ptenA^−^* cells with cAMP, which induces chemotactic competency in a high cAMP concentration gradient [32], is accompanied by up-regulation of *lpten* expression. We therefore conclude that *lpten* plays a similar, but less prominent in pseudopod suppression, motility and chemotaxis role than its homolog *ptenA*, but when overexpressed in the *ptenA^−^* mutant, rescues all of the *ptenA^−^* defects. This raises the question of whether any of the homologs of human *PTEN* might also be induced to function redundantly in cancer cells carrying mutations in *PTEN*.

## Material and Methods

### Strain maintenance, growth and development

The *ptenA^−^* strain DBS0252655 [32] and the parental wild type strain Ax2 [45] were provided by the *Dictyostelium* stock center (http://dictybase.org/StockCenter/StockCenter.html). Methods for growing cells, initiating development and obtaining aggregation-competent amoebae have been described previously in detail [30], [46]–[48]. In brief, development was initiated by washing growth phase cells with buffer and distributing them on filterpads or on HAB04700 nitrocellulose filter pads (Millipore, Billerica, MA, USA) saturated with buffered salts solution (BSS) [49], as previously described [48], [50], [51].

### DNA, RNA purification, cloning and sequencing

Isolation, purification, amplification and sequencing of all the genomic DNA, RNA and cDNA fragments from *D. discoideum* Ax2, mutant strains and plasmids was done as previously described [48]. Plasmids and competent cells were obtained from Life Technologies, (Carslbad CA, USA) [48]. For RNA, recombinant RNasin Ribonuclease (Promega, Madison, WI, USA) was added to inhibit RNA degradation. RNA was additionally purified from residual genomic DNA by using RNAeasyPlus (Qiagen, Ventura, CA, USA) according to the manufacturer's instructions. The primers used in this study are listed in a Table S1. Transformants were generated as described [48] and selected for using either 10 µg/ml Blasticidin S (Enzo Life Science, Farmingdale, NY, USA) and/or 20–70 µg/ml G418 (Sigma-Aldrich, St.Louis, USA). For clonal growth selection of the *ptenA^−^*/*lpten^oe^* and *lpten^−^*/*lpten^oe^* strains, cells were sorted by FACS as described [48].

### Generation of a *Dictyostelium discoideum* knock out strain

**Figure 1:**
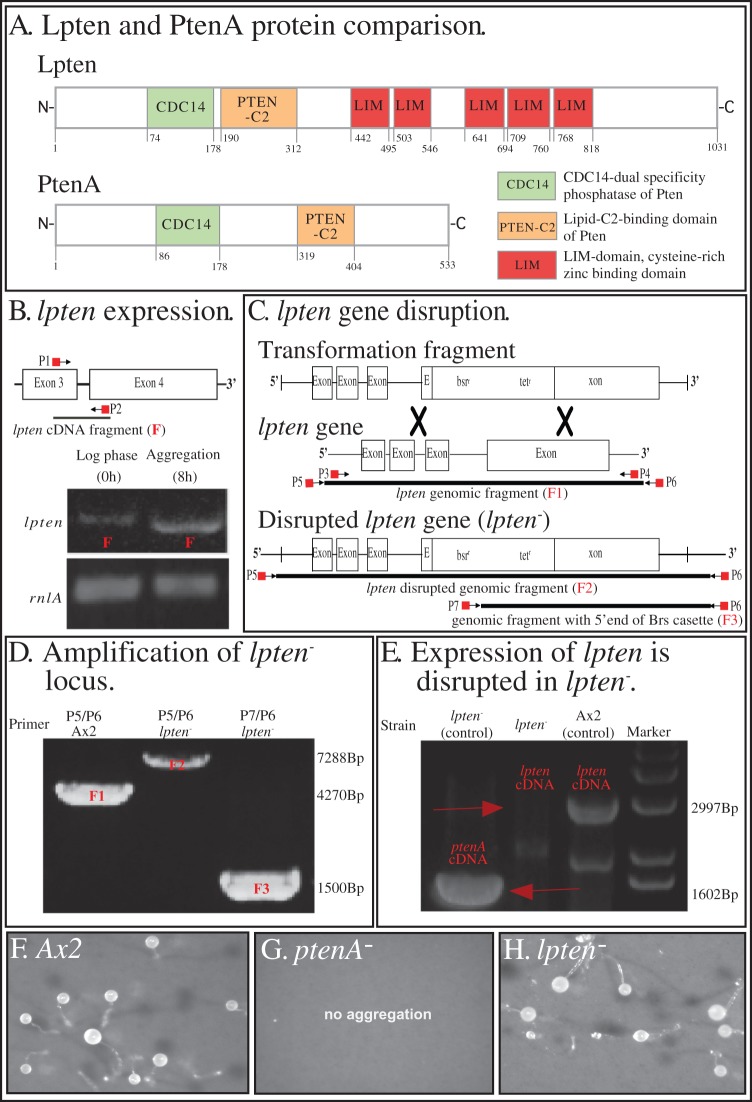
Lpten is a homolog of *ptenA* and an ortholog of human PTEN. *lpten* was disrupted to produce the *lpten* null mutant *lpten^−^*. A. A comparison of Lpten and PtenA. The number of amino acids, the two conserved domains, CDC14 and PTEN-C2, and the LIM domains, are indicated. B. RT-PCR revealed that *lpten* is up-regulated during aggregation. *lpten* expression during development was assessed by RT-PCR and quantified by densitometry. *lpten* expression was up-regulated more than 10 fold. P1and P2 (see Table S1) demark the positions of the primers used to amplify the 300bp *lpten* cDNA fragment (F). RT-PCR of the large subunit ribosomal RNA, *rnlA*, was assessed for comparability of gel loading. C. Scheme for *lpten* disruption. The positions of primers P3 to P7 (see Table S1) are demarcated for amplification of *lpten* in control Ax2 and *lpten^−^* cells, to generate the undisrupted *lpten* genomic fragment F1, the disrupted *lpten* genomic fragment of *lpten^−^*, F2 genomic fragment F2, and a partial *lpten^−^* genomic fragment with a partial *bsr* cassette, F3. D. Verification of *lpten^−^* disruption by PCR. See panel C for the positions of the primers to generate fragments F1, F2 and F3. E. Verification that the *lpten* transcript is missing in the *lpten^−^* mutant using RT-PCR with the primers LptenFW and PtencDNArv for *ptenA*, and PtenAcDNAFW and ptencDNArv to demonstrate the presence of *ptenA* in *lpten^−^*. See Table S1 for description of primers. F. The completion of multicellular morphogenesis by the formation of fruiting bodies in control Ax2 cells. G. The absence of morphogenesis by *ptenA^−^* cells. H. The completion of multicellular morphogenesis by *lpten^−^* cells.

A plasmid was generated that contained a *bsr* resistance cassette containing the gene coding for blasticidin deaminase flanked by *lpten* genomic fragments, as described by Torija et al [52] and diagrammed in Figure 1. In brief, a genomic fragment (F1) (Figure 1C) containing the *lpten* open reading frame and upstream and downstream regions was cloned and incubated in the presence of a PvuII-digested EZTN-plasmid (Epicenter, Madison, WI, USA), which contained a transposon bearing the Blasticidin S resistance marker [53]. Transposase was used to insert the transposon carrying the *bsr* gene into the *lpten*-containing plasmid (Epicentre). Transformed bacteria were selected for by tetracyclin (15 µg/ml) and kanamycin (50 µg/ml). Bacterial colonies bearing the plasmid that had a transposon within the genomic fragment were identified using primer M13, T7 and EZTN-R (Table S1). For *D. discoideum* Ax2 [32], [45] transformation, a fragment of the plasmid carrying the insert containing the *bsr*-resistance cassette close to the 5′ end of the *lpten* coding region was amplified as described by Torija et al. [52], except for the use of Expand Long Template PCR Polymerase (Roche, Indianapolis, IN, USA) [48]. Selection was done with increasing Blasticidin S concentrations. Surviving cells were clonally plated on nutrient plates in the presence of 30 µg/ml Blastidicin S and *Klebsiella aerogenes*. Colonies were harvested using MasterAmp Buccal Swab DNA extraction solution (Epicentre) and subjected to *D. d*iscoideum colony PCR [48], [52].

### Generation of *lpten* overexpression constructs

To obtain an RFP-Lpten fusion, we amplified and cloned the coding region of *lpten* into the extrachromosomal plasmid pDM354 [54]. Cloning and recombination of the *lpten* cDNA were performed as described [48]. Transformed bacterial clones were identified by colony PCR.

### RNA expression analyses

To study expression levels, RT-PCR was performed using the LongRange 2Step RT-PCR Kit (Qiagen) [48]. 2 µg of total RNA, pretreated at 65°C for 5 min, underwent the reverse transcription reaction in a total volume of 20 µl using OligodT primer supplied by the manufacturer. The resulting cDNA was amplified using the Long Range Expand Polymerase Kit (Roche, Indianapolis, IN) and primer P1 and P2 (Table S1, Figure 1). RNA expression levels were quantified under subsaturation conditions as described [55], using the densitometry function of the 2D-DIAS software program [56].

### Analyses of basic cell behavior and chemotaxis

For analyses of basic motility in the absence of chemoattractant and during chemotaxis, cells were harvested from developmental filters at the onset of aggregation, when velocity and chemotactic responsiveness were maximal [57]. For basic behavior in buffer, cells were analyzed on the glass wall of a Sykes-Moore chamber perfused with the buffer BSS according to methods previously described [20], [58]–[60]. The methods for measuring chemotaxis in a Zigmond chamber have also been described in detail elsewhere [60]–[63]. For a “low cAMP concentration” gradient, the source well contained 1 µM cAMP. For a “high cAMP concentration” gradient, the source well contained 10 µM cAMP.

### 2D- and 3D-DIAS analyses of cell behavior

Cell images were digitally acquired using iStopMotion software (Boinx Software, www.boinx.com) and converted to QuickTime format for edge detection, perimeter reconstruction and motion analysis of cell behavior with 2D-DIAS software [64], as previously described [58], [65]. Descriptions of motility and chemotaxis parameters are presented in table S2 [58], [64], [65]. For 3D reconstruction, cells were optically sectioned and analyzed as previously described [20], [58], [59], [66]–[68], except that the resulting QuickTime movies of optical sections were exported into jpeg files and converted into JDIAS movies using an up-graded version of 3D-DIAS ([20], [58], [69], JDIAS 4.1 (Soll et al. 2014 in prep.). The in-focus perimeters were automatically outlined in each optical section using a pixel complexity algorithm [64], [70]. Pseudopods were manually traced.

### Analyses of cell behavior in a natural wave

Natural waves were relayed in populations of cells aggregating on a plastic surface in submerged cultures in 35 mm Petri dishes (Fisherbrand, Pittsburg, PA). Cell behavior was recorded and motion analyzed as previously described [30], [66], [71], [72]. Cell behavior in this case was analyzed using JDIAS 4.1.

### cAMP-pulsing of cells in suspension

Cells were pulsed as described by Hoeller and Kay [32]. In brief, 2×10^7^ cells from a growth culture were washed free of nutrients, shaken in a suspension culture for 1h in buffered salts solution, and subsequently pulsed with 80 nM cAMP at 6 min intervals for 6 h, until aggregate formation was visually observed.

## Results

### *Lpten* is a homolog of *ptenA*

PtenA of *D. discoideum* [28], [29] contains two functionally important and conserved domains, which are present in human PTEN, a CDC14-dual specificity phosphatase (protein cluster COG2453) [73], found in members of the protein tyrosine phosphatase superfamily, and the lipid-C_2_-binding domain, Pten-C2 (PFAM10409) [74] (Figure 1A). A database search of the genome sequence of *D. discoideum* revealed a second ortholog of the human *PTEN* gene, *lpten,* (accession number KF430369), which encodes a protein that also contains the conserved CDC14 dual specificity phosphatase domain and the lipid-C2 binding domain (Figure 1A). In addition, this homolog contains five LIM domains that together contain a total of 38 putative zinc-binding sites (Figure 1A). Because of the LIM domains, we have named this ortholog *lpten*. The amplified cDNA of *lpten* encodes a putative protein of 114 KDa.

### Expression of *lpten*

The *ptenA* expression was previously shown to be low during *D. discoideum* growth and to increase during the preaggregative period of development [75], [76]. A reverse transcriptase-polymerase chain reaction (RT-PCR) was employed to assess *lpten* expression in parental Ax2 cells during growth and at the end of the preaggregative period preceding chemotaxis, using a 300 bp probe (F) that spanned exons 3 and 4, as diagrammed in Figure 1B. *Lpten* was expressed in log phase cells (0 hours) at a very low level and was dramatically up-regulated, approximately ten fold or more in developing cultures at the onset of aggregation (8 hours) (Figure 1B).

### Disruption of *lpten* and mutant rescue

To disrupt *lpten*, Ax2 cells were transformed with an integrative construct, as diagrammed in Figure 1C. Integration was confirmed by PCR (Figure 1D), using primers P5 and P6 to generate fragment F2, as diagrammed in Figure 1C. To further confirm integration, a portion of the disrupted *lpten* gene was amplified with primers P7 and P6, to generate fragment F3 (Figure 1C and D). Sequencing of the product F3 confirmed integration. In contrast to *ptenA^−^* cells, which exhibited a major increase in generation time from 9 to 14 hours [30], [31], *lpten^−^* cells exhibited a generation time of approximately 8 hours, similar to that of the parental strain Ax2 cells. And in contrast to *ptenA^−^* cells, which do not complete aggregation (Figure 1G), *lpten^−^* cells underwent aggregation and multicellular morphogenesis, forming fruiting bodies (Figure 1H). The *lpten^−^* mutant was rescued by transformation with a plasmid containing *lpten* fused to *rfp* under the regulation of the *actin 15* promoter. The complemented mutant strain, *lpten^−^/lpten^oe^*, grew with the same generation time as parental Ax2 cells, aggregated and formed fruiting bodies (data not shown). Therefore, deleting *lpten* resulted in no measurable growth or obvious developmental defect.

### *lpten* ^−^ cells exhibit a minor defect in basic cell motility

**Figure 2:**
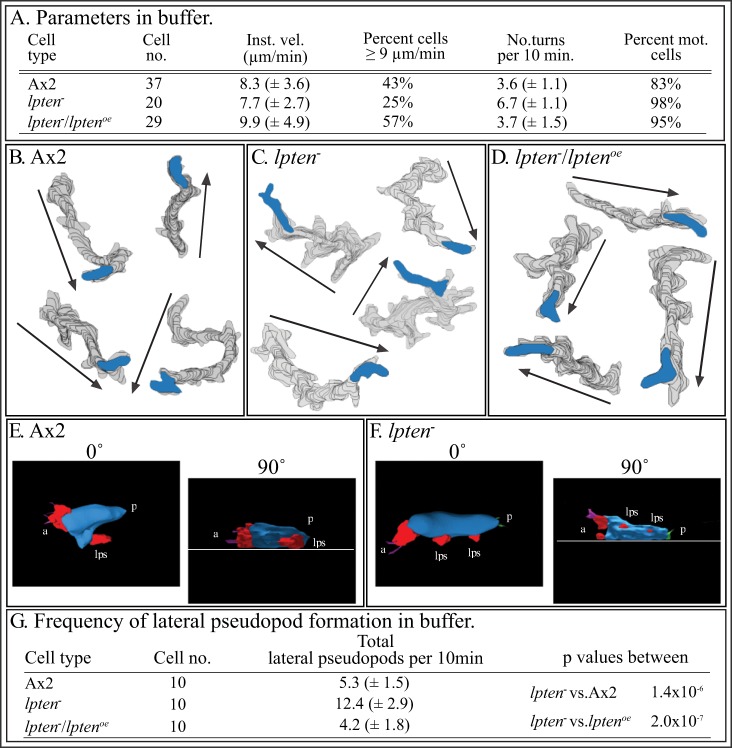
*lpten^−^* cells translocating in buffer in the absence of chemoattractant exhibit defects in velocity, turning and the suppression of lateral pseudopod formation. Cells were analyzed in a perfusion chamber through which buffer without attractant was pumped. A. 2D motility parameters of Ax2, *lpten^−^* and *ptenA^−^/lpten^oe^* cells assessed with 2D-DIAS software. B, C, D. 2D-DIAS reconstructions of cell perimeters to generate tracks. Arrows denote net direction, and the blue-filled perimeters represent the last cell positions in the tracks. E, F. 3D-DIAS reconstructions at 0° (top view) and 90° (side view) of representative Ax2 and *lpten^−^* cells, respectively, denoting pseudopods (red). Note that the multiple lateral pseudopods formed by *lpten^−^* cells, were primarily off the substrate. a, anterior end of cell; p, posterior end of cell; lps, lateral pseudopod. G. 2D analysis of lateral pseudopod formation. Inst. vel., instantaneous velocity; No. turns per 10 min., number of turns per 10 minutes; Percent mot. cells, percent motile cells. Parameters are presented as the means ± standard deviations. T-test was used to determine p values. Parameters are defined in Table S2.

Using computer-assisted 2D and 3D reconstruction and motion analysis systems, we previously demonstrated that aggregation-competent *ptenA^−^* cells perfused with a K^+^-based buffer [30], [77] lacking chemoattractant exhibited a 50% decrease in velocity, a major increase in turning and a four-fold increase in lateral pseudopod formation [30]. Using the same computer-assisted methods, we found that *lpten^−^* cells exhibited basic behavioral defects similar to those of *ptenA^−^* [30] cells, in instantaneous velocity, percent cells with velocities ≥9 µm per minute and turning (Figure 2A). However, the defects although significant (p value <0.05), were less pronounced. The defects were evident in comparisons of computer-reconstructed cell perimeter tracks (Figure 2C), when compared to those of parental Ax2 cells (Figure 2B) or complemented *lpten^−^/lpten^oe^* cells (Figure 2D). 3D reconstructions performed with 3D-DIAS software [58], [64], [70] revealed that *lpten^−^* cells, like *ptenA^−^* cells [30], formed lateral pseudopods, which initiate turns, at frequencies higher than Ax2 and *lpten^−^/lpten^oe^* (Figure 2E, G and F, respectively). 2D measurements of the frequency of lateral pseudopods formed by reconstructed control (Ax2) cells, *lpten^−^* cells and *lpten^−^/lpten^oe^* cells, supported this conclusion (Figure 2G). The frequency of the mutant was over twice that of Ax2 and *lpten^−^/lpten^oe^* cells (Figure 2G).

### *lpten^−^* cells undergo normal cAMP chemotaxis, but still are defective in suppressing lateral pseudopod formation

**Figure 3:**
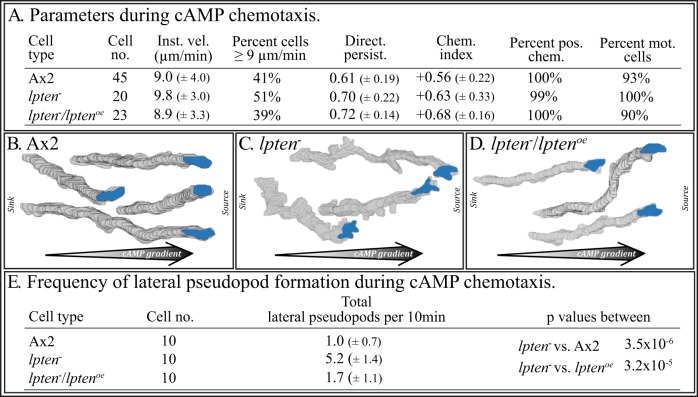
*lpten^−^* cells undergo normal chemotaxis in the low cAMP concentration gradient generated in the estimated for that of the natural wave. The gradient was generated in BSS buffer, in which K^+^ and Na^+^ are the facilitating cations. *lpten^−^* cells, however, are still defective in suppressing lateral pseudopod formation. A. 2D motility and chemotaxis parameters, assessed by 2D-DIAS software of Ax2, *lpten^−^* and *lpten^−^/lpten^oe^* cells undergoing chemotaxis in a low cAMP concnetration gradient. B, C, D. 2D-DIAS-reconstructed perimeter tracks of representative cells. The large arrows at panel bottoms denote the net direction of the increasing cAMP gradient. “Sink”, trough with buffer alone; “Source”, trough with buffer plus 1 µM cAMP. E. 2D analysis of lateral pseudopod formation. Direct. Persist, directional persistence; chem. index, Chemotactic Index (CI); Percent pos. chem., percent cells with a positive CI. See legend to Figure 2 for additional definitions and details. Parameters are defined in Table S2.

We previously demonstrated that *ptenA^−^* cells undergoing positive chemotaxis in a spatial gradient of cAMP generated in the concentration range estimated for the front of a natural wave [44] (i.e., low cAMP concentration gradient), exhibited approximately a 50% decrease in velocity and a similar decrease in directional persistence [30]. Moreover, the efficiency of chemotaxis, measured by the chemotactic index (CI), was reduced by more than 50% [30]. Under identical conditions, *lpten^−^* cells moved with an average velocity, average directional persistence, average chemotactic index (CI) and percent cells with a positive CI, statistically indistinguishable, using the student T-test (data not shown), from that of parental Ax2 cells and complemented *lpten^−^/lpten^oe^* cells (Figure 3A). Computer-reconstructed perimeter plots revealed similar directionality up a low cAMP concentration gradient (Figure 3B, C, D), but *lpten^−^* cells were still defective in suppressing lateral pseudopod formation (Figure 3C), as is evident when the perimeter tracks of mutant cells are compared to those of Ax2 (Figure 3B) and *lpten^−^/lpten^oe^* cells (Figure 3D). This was demonstrated in the analysis of the frequency of lateral pseudopod formation (Figure 3E). 3D reconstructions revealed that the majority of the excess lateral pseudopods in cAMP gradients were formed by *lpten^−^* cells off the substratum (data not shown), as was evident in buffer in the absence of cAMP (Figure 2A, F). Lateral pseudopods that contact the substratum force turns [78]. Hence, the formation of excess lateral pseudopods off the substratum did not result in a significant increase in turning, which may explain why there was no significant decrease in the CI.

### Generating a *ptenA* ^−^ */lpten^oe^* strain

**Figure 4:**
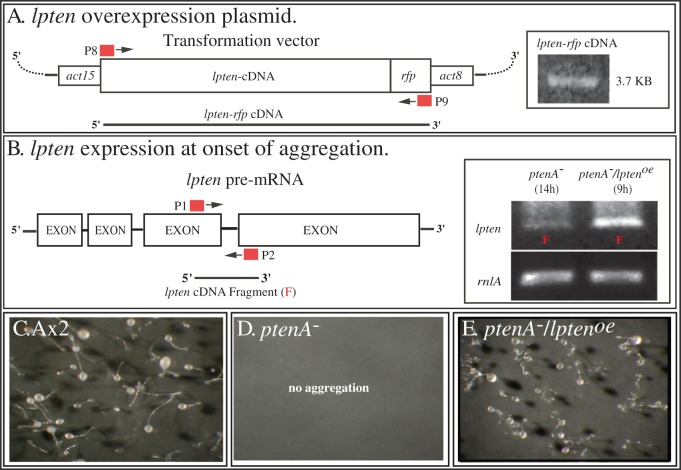
Overexpressing *lpten^−^* in the *ptenA^−^* mutant. A. The transformation vector used to generate strains *ptenA^−^/lpten^oe^*, in which *lpten* is under the regulation of the *actin 15* (*act15*) promoter, fused in frame at the 3′ end to the red fluorescent protein gene (*rfp*) and terminating with a 3′ *actin 8* gene sequence. The positions of the primers P8 and P9, for generating the *lpten-rfp* cDNA, are denoted. Insert shows verification of the *lpten-rfp* cDNA by PCR. B. *lpten* is expressed in *ptenA^−^/lpten^oe^* cells at levels more than 10 times that in the parent *ptenA^−^* mutant. The positions of the primers (P1, P2) for RT-PCR of the 300 bp *lpten* fragment (F) are denoted. In the insert to the right of panel B, RT-PCR products of chemotactically responsive *ptenA^−^* and *ptenA^−^/lpten^oe^* cells reveals overexpression of *lpten* in the latter. Densitometry measurements revealed>10 fold overexpression. C. Fruiting body formation in Ax2 cultures. D. The absence of fruiting body formation in *ptenA^−^* cultures. E. Fruiting body formation in *ptenA^−^/lpten^oe^* cultures. See Table S1 for description of primers.

The similarities between the strong behavioral defects of the *ptenA^−^* mutant and the weaker defects of the *lpten^−^* mutant, suggested that the two homologs may play overlapping roles in basic cell motility and chemotaxis. We therefore tested whether overexpressing *lpten* in the *ptenA^−^* mutant would partially alleviate, or even rescue, the severe defects exhibited by *ptenA^−^* cells. The *ptenA^−^* mutant was transformed with an expression plasmid in which the cloned *lpten* coding region was placed under regulation of the strong *actin15* promoter and fused in frame at its 3′ end with red fluorescent protein (*rfp*) [79] (Figure 4A). Expression of the entire 3.7 Kb *lpten^−^ rfp* mRNA was verified using RT-PCR with primers P8 and P9 (Figure 4A, Table S1). Upon achieving chemotactic responsiveness, aggregation-competent cells of the transformed line *ptenA^−^/lpten^oe^* expressed approximately 10 times as much *lpten* mRNA as the untransformed *ptenA^−^* mutant (Figure 4B, inserted box). Overexpression of *lpten* rescued the developmental defects of the *ptenA^−^* mutant, resulting in normal aggregation (data not shown) and the formation of normal fruiting bodies (compare Figures 4C, D and E).

### Overexpression of *lpten* rescues the basic behavioral and chemotactic defects of *ptenA* ^−^ cells

**Figure 5:**
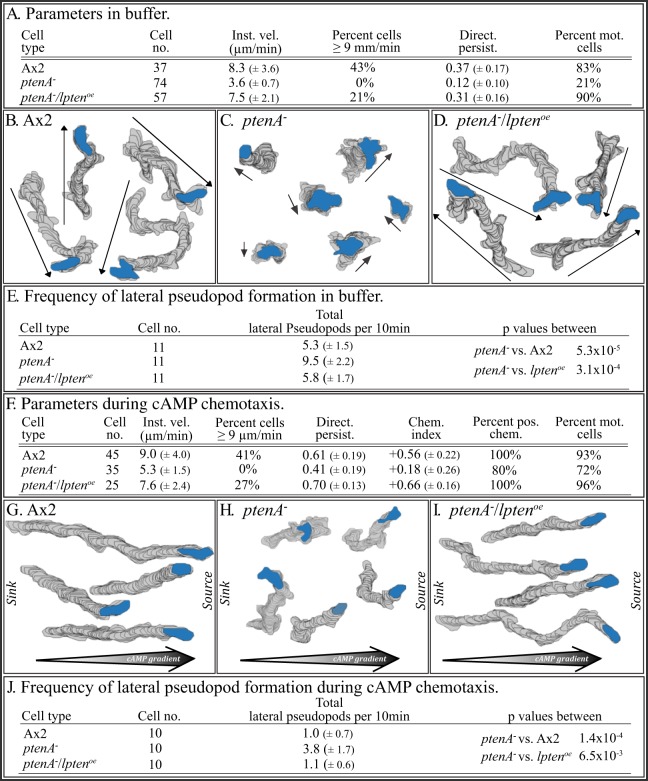
Overexpression of *lpten* rescues the basic behavioral defects of *ptenA^−^* cells that are translocating in buffer, and both the behavioral and chemotactic defects in a cAMP gradient generated in the concentration range of the natural wave. A. 2D motility parameters of cells translocating in buffer, assessed by 2D-DIAS software. B, C, D. 2D-DIAS reconstructions of perimeter tracks of Ax2, *ptenA^−^* and *ptenA^−^/lpten^oe^* cells, respectively, translocating in buffer. E. 2D analysis of lateral pseudopod formation in buffer. F. 2D motility and chemotaxis parameters assessed by 2D-DIAS software during chemotaxis in a low cAMP concentration gradient. G, H, I. Perimeter tracks of cells in a low cAMP concentration gradient. J. 2D analysis of lateral pseudopod formation during chemotaxis in a low cAMP concentration gradient. See the legend to Figure 2 for explanations of panels A through E, and the legend to Figure 2 and 3 for explanations of panels F through J.

As previously demonstrated [29], [30], cells of the *ptenA^−^* mutant originally generated by Iijima and Devreotes [29], did not undergo morphogenesis on filter pads saturated with a K^+^-based buffer. Furthermore, when incubated on pads saturated with K^+^-based buffer to attain chemotactic competence and then assessed for basic motile behavior on the glass surface of a chamber perfused with K^+^-based buffer lacking cAMP, these cells crawled at less than half the average velocity of control cells and with less than half the directional persistence. These same characteristics were observed in the *ptenA^−^* mutant used here, which was generated by Hoeller and Kay [32] (Figure 5A). The abnormal behavior of *ptenA^−^* cells was obvious, when computer-reconstructed perimeter tracks of control and *ptenA^−^* cells translocating in buffer were compared (Figure 5B and C). The *ptenA^−^* cells translocating in buffer also formed lateral pseudopods at frequencies close to twice that of control cells (Figure 5E), as was the case for the *lpten^−^* strains (Figure 2G). Overexpression of *lpten* in *ptenA^−^/lpten^oe^* cells rescued every motility defect in the basic behavior of cells migrating in buffer in the absence of chemoattractant (Figure 5A), resulting in normal perimeter tracks (Figure 5D) and restored suppression of lateral pseudopod formation (Figure 5E).

And, as previously demonstrated, *ptenA^−^* cells exhibited the same motility defects in a low cAMP concentration gradient, as they did in buffer alone, as well as a dramatic decrease in chemotactic responsiveness [29], [30] (Figure 5F, H, J). Overexpression of *lpten* in *ptenA^−^/lpten^oe^* cells rescued every motility and chemotaxis defect (Figure 5F), resulting in directed motility tracks up a gradient (Figure 5I) and restored suppression of lateral pseudopod formation (Figure 5J).

### Overexpression of *lpten* rescues the *ptenA* ^−^ defect in natural aggregation

**Figure 6:**
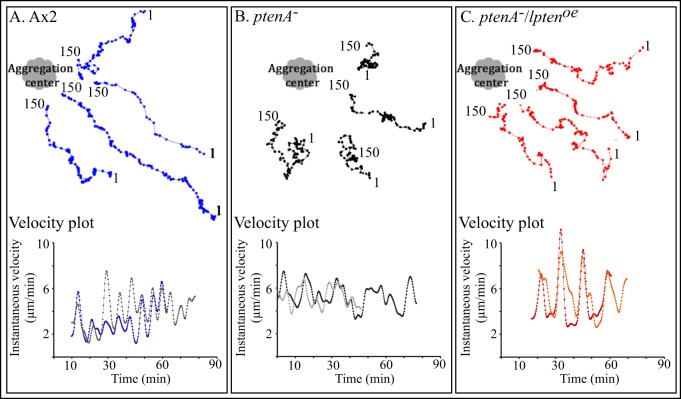
Overexpression of *lpten* rescues the behavioral defects exhibited by homogeneous populations of *ptenA^−^* cells undergoing chemotaxis in natural aggregation territories in submerged cultures on glass. A, B, C. The centroid tracks of four neighboring cells representative of the general behavior of Ax2, *ptenA^−^* and *ptenA^−^/lpten^oe^* populations, respectively, are presented in relation to the aggregation centers of Ax2 and *ptenA^−^/lpten^oe^* cells, and the interpreted aggregation center of *ptenA^−^*, deduced retrospectively by the direction of net translocation of groups of cells, in the upper half of each panel. The first (1) and last (150) centered in the centroid tracks are noted. In lower half of each panel, the velocity plots are presented for two respective cells. For normal cells, the peaks of velocity have been shown to correlate with the front of each relayed natural wave.

Finally, as Wessels et al. [30] demonstrated, *ptenA^−^* cells [32] are defective in natural aggregation. In a natural aggregation territory, parental Ax2 cells moved in a highly directed (Figure 6A) and cyclic fashion towards aggregation centers, increasing velocity in the front of each relayed, outwardly moving, non-dissipating wave of cAMP (velocity plots for two representative neighboring cells in the lower portion of Figure 6A). This resulted in centroid tracks that were directed at the source of chemotactic waves. Cells decreased velocity in the back of each wave, then reassessed directionality at the onset of the front of each wave, adjusting for deviations in direction during the translocation phase [48], [51]. To assess aggregation centers in *ptenA^−^* cell populations, which do not complete normal aggregation, we retrospectively identified the point in each putative aggregation territory to which cells made net directional progress. Although the *ptenA^−^* cells on average did make net progress towards the interpreted aggregation centers (Figure 6B, upper portion) and exhibited cyclic behavior (Figure 6B, lower portion), their centroid tracks were stunted and far less directional (i.e., less oriented on average in the direction of the interpreted aggregation center) (Figure 6B, upper portion) and cycling was more erratic (Figure 6B, lower portion). *ptenA^−^* cells tended to undergo far more directional changes than control cells, resulting from sharp turns away from the interpreted aggregation center. Overexpression of *lpten* in *ptenA^−^/lpten^oe^* cells restored normal behavior in a natural aggregation territory (Figure 6C). Cells surged in the front of each wave (Figure 6C, lower portion) and moved in a relatively directed fashion, with far fewer sharp turns, towards the aggregation center (Figure 6C, upper portion), in a manner similar to that of parental Ax2 cells (Figure 6A, upper portion).

### Pulsing *ptenA* ^−^ cells up-regulates *lpten* and rescues chemotaxis in high, but not low, cAMP concentration gradients

In performing this study, one apparent controversy had to be resolved. In three previous studies of *ptenA^−^* cell behavior [29], [30], [32], similar defects in velocity were described, but there was a lack of consensus on the capacity of mutant cells to assess a spatial gradient of cAMP generated *in vitro*. In all three studies, data were provided for cells that were induced to acquire chemotactic competence by a similar method of pulsing with cAMP [80], [81] (Table 1), rather than incubating them on pads saturated with buffer, as performed for the experiments reported here (Table 1). All three studies employed buffers in the *in vitro* chemotaxis assays (Table 1) that contained concentrations of cations that facilitated cAMP chemotaxis [48], [51], [59], [77]. However, the studies differed in the concentration range of the cAMP gradients used. Iijima and Devreotes [29] analyzed responsiveness in a gradient of cAMP generated by releasing 1µM cAMP from a micropipette (low cAMP concentration gradient). They observed that *ptenA^−^* cells exhibited reduced velocity and a loss of chemotactic orientation (Table 1). Wessels et al. [30] analyzed chemotactic responsiveness in a gradient generated in a chamber [61], [63], in which the source well was filled with 1 µM cAMP (low cAMP concentration gradient). They observed a 40% reduction in velocity and a 60% reduction in the chemotactic index (Table 1). The gradients in these two studies were estimated, using the methods of Postma and Van Haastert [82], to be approximately 0.5 and 0.4 nM per µm and in the concentration range of the gradient estimated for the front of a natural wave in an aggregation territory (i.e., 1 µM at the peak of each wave and less than 0.01 µM at the trough) [44]. In contrast, Hoeller and Kay [32] analyzed responsiveness in a cAMP gradient generated by releasing 10 µM cAMP from a micropipette. The gradient generated was estimated to be approximately 5 nM per µm, steeper and in a concentration range 10 times higher than gradients tested by Iijima and Deveotes [29] and Wessels et al., [30]. Hoeller and Kay [32] observed no difference in chemotactic efficiency between Ax2 and *ptenA^−^* cells, but did report a defect in velocity.

**Table 1:** Chemotactic behavior of *ptenA^−^* cells pulsed with cAMP to achieve chemotactic competence: a comparison of four different studies involving either “low cAMP concentrations” in the estimated range for natural cAMP waves or “high cAMP concentration gradients”, at concentrations 10 times that of natural cAMP waves.

Study	Mutant origin (references)^ a^	Conditions for the induction of chemotactic competence	Cation content of buffer employed in the chemotaxis assay^b^	Concentration of cAMP at source and concentration range^c^	Method assessing chemotactic efficiency	*ptenA^−^*velocity	*ptenA^−^*chemotaxis	Natural aggregation and fruiting body formation
Iijima and Devreotes, 2002 [29]	[29]	100 nM cAMP pulses for 6 hours at 6 min intervals in DB buffer	DB: 15 mM Na^+^; 0.2 mM Ca^2+^; 2 mM Mg^2+^	1 µM	Release from micropipette	Greatly reduced	Lost	Lost
Hoeller and Kay, 2007 [32]	[32]	Starvation for 1 hr, 70–90 nM cAMP pulse for 3–5 hours at 6 min intervals in KK2 buffer	KK2: 24 mM K^+^; 0.1 mM Ca^2+^	10 µM	Release from micropipette	Normal	Normal	Lost
Wessels et al., 2007 [30]	[29]	50 nM cAMP pulsed for 6 hr at 6 min intervals in BSS buffer	BSS: 45 mM Na^+^ plus K^+^; 2.5 mM Mg^2+^	1µM	Gradient chamber	Normal	Near normal	Lost
This study	[32]	Starvation for 1 hr, 80 nM cAMP pulses for 5 hours at 6 min intervals in BSS buffer	BSS: 45 mM Na^+^ plus K^+^; 2.5 mM Mg^2+^	1) 1 µM	1) Gradient chamber	Reduced 40%	Lost	Lost
				2) 10 µM	2) Gradient chamber	Normal	Normal	Not tested

a. Reference [29]: Iijima and Devreotes, 2002; reference [32]: Hoeller and Kay, 2007; reference [30], Wessels et al., 2007.

b. Complete composition of buffers used in chemotaxis assay. DB buffer: 5 mM Na_2_HPO_4_, 5 mM NaH_2_PO_4,_ 2 mM MgSO_4_, 0.2 mM CaCl_2_ (15 mM Na^+^, 0.2 mM Ca^2+^). KK_2_ buffer: 3.9 mM K_2_HPO_4_, 16.5 mM KH_2_PO_4_, 2 mM Mg SO_4_, 0.1 mM CaCl_2_ (24.3 mM K^+^, 0.1 mM CA^2+^, 0.1 mM Ca^2+^). BSS buffer: 20 mM KH_2_PO_4_, 5 mM NA_2_HPO_4_, 20 mM KCL, 2.5 mM MgCl_2_ (45 mM K^+^/Na^+^, 0 mM Ca^2+^ [77].

c. When the source of the cAMP gradient was 1 µM, it generated a “low cAMP concentration gradient”, in the concentration range estimated for the natural wave [44], and when it was 10 µM, it generated a high cAMP concentration gradient 10 times that estimated for the natural.

**Figure 7:**
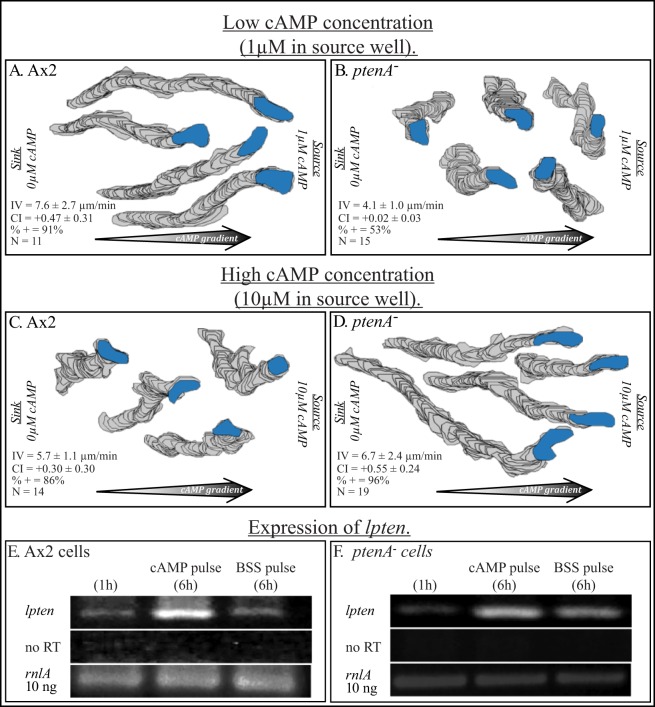
*ptenA^−^* cells pulsed in suspension with cAMP to induce chemotactic competence are defective in assessing the direction of a low cAMP concentration gradient in the range of a natural wave, but they can efficiently assess the direction of a cAMP gradient in a concentration range 10 times higher. Pulsing *ptenA^−^* cells with cAMP also up-regulates *lpten*. A, B. 2D-DIAS reconstructions of perimeter tracks of Ax2 and *ptenA^−^* cells, respectively, in a low cAMP concentration gradient, generated by adding 1 µM cAMP to the source well of the gradient chamber. Motility and chemotaxis parameters assessed by 2D-DIAS software are presented in the lower left hand corner of each panel. C, D. Perimeter tracks of representative Ax2 and *ptenA^−^* cells, respectively, in a high cAMP concentration gradient, generated by adding 10 µM cAMP to the source well of the gradient chamber. Motility and chemotaxis parameters are displayed in the lower left corner of each panel. E, F. Up-regulation of *lpten* expression in cAMP pulsed Ax2 and *ptenA^−^* cells, respectively. In each strain, cells were analyzed by RT-PCR using primers P1 and P2 (Table S1), prior to cAMP pulsing (1 hr), after cAMP pulsing for six hours (6 hr) and after cAMP pulsing with buffer for six hours (6 h). The constitutively expressed large subunit ribosomal RNA (*rnlA*) was assessed for comparability (see Figure 1 and 4). No RT, no reverse transcriptase added; IV, instantaneous velocity; CI, chemotactic index; %+, percent cells with a positive CI; N, number of cells assessed. Parameters in panels A, B, C and D are defined in Table S2.

These results suggested to us the possibility that pulsing *ptenA^−^* cells with cAMP might up-regulate expression of *lpten* to a level that provides them with the capacity to assess the gradient in the high cAMP concentration range, but not the gradient in the low cAMP concentration range. To explore this hypothesis, we analyzed the behavior of cAMP-pulsed parental cells and cells of the *ptenA^−^* strain of Hoeller and Kay [32], in gradients generated in a gradient chamber [58], [61], [63]. Behavior was analyzed in low cAMP concentration gradient, in which the source well contained 1 µM cAMP (Figures 7A and B) and in a high cAMP concentration gradient, in which the source well contained 10 µM cAMP (Figures 7C, D).

In the low cAMP concentration gradient, cAMP-pulsed parental Ax2 cells underwent chemotaxis with high velocities and high chemotactic indices (Figure 7A). And as previously reported [29], [30] and shown here in Figure 5, both the instantaneous velocity and the CI of *ptenA^−^* cells were dramatically reduced (Figure 7B). In a high concentration gradient, cAMP-pulsed parental Ax2 cells underwent chemotaxis, but velocity was reduced by 25% and chemotactic efficiency (C.I.) by 35% (Figure 3C), reductions similar to those previously reported by us using the same conditions thirty years ago [63]. However, in a high cAMP concentration gradient, *ptenA^−^* cells moved with a chemotactic index similar to that of Ax2 cell in a low concentration gradient, with slightly reduced velocity, as previously reported by Hoeller and Kay [32].

To test whether cAMP pulsing caused an increase in *lpten* expression in *ptenA^−^* cells, the level of the *lpten* transcript was compared between Ax2 and *ptenA^−^* cells by RT-PCR prior to pulsing (1 h), after six hours of pulsing with cAMP and after 6 hours of pulsing with buffer alone. Both in Ax2 cells (Figure 7E) and *ptenA^−^* cells (Figure 7F), cAMP pulsing up-regulated *lpten* expression at least 5 fold over that of the initial vegetative cell preparation (0 h). Pulsing *ptenA^−^* cells with buffer alone also up-regulated *lpten* expression, but to a lesser degree than pulsing with cAMP (Figure 7E, F). These results demonstrate that pulsing with cAMP up-regulates *lpten* expression, and, by correlation, may explain why cAMP-pulsed *ptenA^−^* cells can undergo chemotaxis in a high cAMP concentration gradient. The increased levels of expression of *lpten^−^* in cAMP-pulsed Ax2 and *ptenA^−^* cells were still several fold lower than the levels attained in strain *ptenA^−^/lpten^oe^* cells developed on pads (data not shown). This may explain why pulsing does not rescue the chemotaxis defect in a low cAMP concentration gradient.

## Discussion

Mutations in the human *PTEN* gene are the most common of the tumor suppressor genes associated with human cancer [2], [3], [12], [83]. Interestingly there are two additional *PTEN* homologs, *TPTE* and *TPIP*located on different chromosomes, and a secreted *PTEN* [12], [13], [15]–[18], as well as a pseudogene of *PTEN*, *PTENP1* [17]. However, there have been, to our knowledge, no reported studies to test whether any of the human *PTEN* homologs, when overexpressed, can rescue the behavioral defects caused by a human mutant *PTEN* cell, a question relevant to cancers that involve this mutation.

Here, we report for the first time that *D. discoideum* contains not only the human *PTEN* ortholog *ptenA*, as previously demonstrated [28], [29], but also a second ortholog, *lpten*. Both PtenA and Lpten contain the two conserved domains of human PTEN, the dual-specificity phosphatase domain and PTEN-C2, the lipid-C2-binding domain. In addition, Lpten contains five LIM domains, which presumably play a role in protein-protein interactions [84], [85]. Both *ptenA* and *lpten* are up-regulated in the period of the *D.discoideum* developmental program following the onset of starvation and preceding aggregation. Deletion of *ptenA* causes major defects in chemotaxis and development [29], [30], [32]. The *ptenA^−^* cells cannot undergo natural chemotaxis, aggregation or morphogenesis. However, deletion of *lpten* does not block aggregation or development, and does not decrease the efficiency of chemotaxis in a gradient of cAMP generated *in vitro* in the concentration range of the cAMP gradient in the front of the naturally relayed cAMP wave. Deletion of *lpten* does, however, affect the suppression of lateral pseudopod formation, which is also the case for the *ptenA^−^* mutant. The mutant phenotype of *lpten^−^*, therefore, exhibits a weak phenocopy of *ptenA^−^*.

Here we have considered the possibility that there may exist at least parallel functions, or partial redundancy of Pten and Lpten. This has led us to test whether overexpressing *lpten* in a *ptenA^−^* mutant might rescue the defects of the *ptenA^−^* mutant. We therefore placed the coding region of *lpten* under the control of the strong *actin 15* promoter in a plasmid and introduced it into the *ptenA^−^* mutant to create *ptenA^−^/lpten^oe^*. Aggregation-competent cells of *ptenA^−^/lpten^oe^* expressed the *lpten* transcript at over 10 times the level observed in wild type or *ptenA^−^* cells, and close to two orders of magnitude higher than in vegetative cells. We found that overexpression rescued every developmental, cell motility and chemotaxis defect exhibited by mutant *ptenA^−^* cells [30]. The rescued *ptenA^−^* defects included the following: 1) a prolonged preaggregative period; 2) abnormal or no aggregation in submerged cultures or on a filter pad substrate; 3) lack of a multicellular developmental program, including lack of fruiting body formation; 4) decreased velocity during basic motile behavior in buffer alone, or during chemotaxis in a shallow gradient of cAMP generated in the concentration range estimated for the front of a natural wave [44] 5) an increased frequency of lateral pseudopod formation and turning; 6) a dramatic decrease in chemotactic efficiency in a low cAMP concentration gradient, in the estimated range for the front of the natural wave; and 7) a dramatic decrease in orientation in the front of relayed cAMP waves in a natural aggregation territory. The complete rescue of the *ptenA^−^* mutant phenotype by overexpression of the homolog *lpten*, raises the possibility that overexpressing a human *PTEN* homolog might suppress the behavioral defects of a mutant *PTEN* thus suppressing tumorigenesis.

## Supporting Information

Table S1 **Primers used in this study.**
(PDF)

Table S2 **2D-DIAS parameters.**
(PDF)
