# Supplementary material for: PTEN Redundancy: Overexpressing lpten, a Homolog of Dictyostelium discoideum ptenA, the Ortholog of Human PTEN, Rescues All Behavioral Defects of the Mutant ptenA−
Source: PLoS One. 2014 Sep 23;9(9):e108495. doi: 10.1371/journal.pone.0108495 (PMC4172592; doi:10.1371/journal.pone.0108495)
Supplement: Table S1 — Primers used in this study. (PDF) [file pone.0108495.s001.pdf]

Supplemental Table S1. Primers used in this study.

Primer	Sequence	Primer designation
lptenExFw	5’- ATCAAGTACAAATAATACTTTTGAAATC-3’	P1
lptenExRV	5’- ATTTCATTATTTTCAAATACACAATCAC-3’	P2
lptengenomFW	5’- AGAAACGACCAAAATTTTACATC-3’	P3
lptengenomRv	5’- ATTTTAATGAAATAAAATTATCAATA-3’	P4
lptengenomicFw1	5’-ATTTTTGTTTCATCTTAATCTGCCATTATTTGTTTGTCACAACATATCATTTTAGCAAA CAATAG-3’	P5
lptengenomicRv1	5’- TCTTTTGATGTACCATAAAACGAATTTCCCATACTACC-3’	P6
EZTN-R	5’- GCCAATATGCGAGAACACCCGAG-3’	P7
lptenFW	5’- ATGGACTTAGTAAGAAAAATGG-3’	P8
mRFPrv	5’- TGCACCTGTTGAATGTCTACCTTCTG-3’	P9
IG7-FP	5’- TTACATTTATTAGACCCGAAACCAAGCG-3’	Figure 1B,<br>Amplification of *rnlA*
IG7-RP	5’- TTCCCTTTAGACCTATGGACCTTAGCG-3’	Figure 1B,<br>Amplification of *rnlA*
ptenAcDNAFw	5’- ATGAGTAATTTATTAAGAGTTGC-3’	Figure 1E<br>Amplification of *ptenA*
ptencDNArv	5’- ACTTGAGCTATTTGAAGAAG-3’	Figure 1E<br>Amplification of *ptenA*
M13Rv	5’- CAGGAAACAGCTATGA-3’	Methods,<br>Colony PCR to obtain

		lpten-*bsr*-plasmid
T7	5’- GTAATACGACTCACTATAGGG-3’	Methods,<br>Colony PCR to obtain *lpten*-*bsr*-plasmid
lptenFW	5’- TGGTGGATCAAAATCCAGAAAATGTAG-3’	Methods, Colony PCR *D.d.*
lptenRV	5’- TAAGTTGAAGTATTTGTTGTAGTTGT-3’	Methods, Colony PCR *D.d.*
lptenFWEntry	5’- caccATGGACTTAGTAAGAAAAATGG-3’	Methods, Subcloning of aggregation comptent *lpten* cDNA
lptencDNArv	5’- CACTTTTTATTTTTAATATTGATAATTTTATTTCAT-3’	Methods, Subcloning of aggregation comptent *lpten* cDNA
lptenFwseq1	5’- ATGGGTTTTCCTAGTGAATCATTGG-3’	Sequencing *lpten*
lptenFwseq2	5’- ATGTGGTTGGATCAAAATCC-3’	Sequencing *lpten*
lptenFwseq3	5’- TGGTTGTGAACCATATTTATCAATTGTTCAACAAGG-3’	Sequencing *lpten*
lptenFwseq4	5’- AGCACATAAAGATTTAAAACATTTCC-3’	Sequencing *lpten*
lptenFwseq5	5’- ATGTAATCTTCCTATAACTGCAACTGATCC-3’	Sequencing *lpten*
lptenFwseq6	5’- ATGAAACCCCAATGGATCC-3’	Sequencing *lpten*
lptenFwseq7	5’- TGAAAAATCATCATCATCATTATCC-3’	Sequencing *lpten*
