# Supplementary material for: PTEN Redundancy: Overexpressing lpten, a Homolog of Dictyostelium discoideum ptenA, the Ortholog of Human PTEN, Rescues All Behavioral Defects of the Mutant ptenA−
Source: PLoS One. 2014 Sep 23;9(9):e108495. doi: 10.1371/journal.pone.0108495 (PMC4172592; doi:10.1371/journal.pone.0108495)
Supplement: Table S2 — 2D-DIAS parameters. (PDF) [file pone.0108495.s002.pdf]

Supplemental Table S2. 2D-DIAS parameters.

Parameter	Computation	Reference
Instantaneous velocity (Inst.vel.µm/min)	The center of the cell, the centroid was calculated. The distance over time was computed using the central difference method. Image frames were taken at a 4 second interval and the distance taken between each corresponding consecutive pair of centroids over a ten minute period.	[1]
Percent (%) cells ≥ 9 µm per minute	Instantaneous velocities ≥ 9 µm per minute were averaged.	[2]
Directional persistence (Direct.persist.)	Net distance between the first and last centroid of a centroid track over ten minutes period divided by the summed distances between consecutive centroid positions of the track.	[1]
Number (No) of turns per 10 min.	A turn occurred if a newly extended pseudopod redirected subsequent migration during a 10 min period.	[3]
Percent motile cells	Proportion of cells in a population with an instantaneous velocity ≥ 3µm/min	[1]
Chemotactic Index (Chem.index)	The net distance traveled by a cell in the direction of the source of chemoattractant divided by the total distance traveled over a 10 minute period.	[1]
Percent positive (pos.) chemotaxis	Proportion of cells in a population with a chemotactic index greater than 0.	[1]

References

1. Soll, D.R. (1995). The use of computers in understanding how animal cells crawl. Int Rev Cytol *163*, 43-104.

2. Lusche, D.F., Wessels, D., and Soll, D.R. (2009). The effects of extracellular calcium on motility, pseudopod and uropod formation, chemotaxis, and the cortical localization of myosin II in *Dictyostelium discoideum.* Cell Motil. Cytoskeleton *66*, 567-587.

3.Varnum-Finney, B., Edwards, K.B., Voss, E., and Soll, D.R. (1987). Amebae of *Dictyostelium discoideum* respond to an increasing temporal gradient of the chemoattractant cAMP with a reduced frequency of turning: evidence for a temporal mechanism in ameboid chemotaxis. Cell Motil Cytoskeleton *8*, 7-17.
